# Estimated Residential Exposure to Agricultural Chemicals and Premature Mortality by Parkinson’s Disease in Washington State

**DOI:** 10.3390/ijerph15122885

**Published:** 2018-12-16

**Authors:** Mariah Caballero, Solmaz Amiri, Justin T. Denney, Pablo Monsivais, Perry Hystad, Ofer Amram

**Affiliations:** 1 Department of Biology, Vassar College, Undergraduate Student, Poughkeepsie, NY 12604, USA; macaballero@vassar.edu; 2 Department of Nutrition and Exercise Physiology, Elson S. Floyd School of Medicine, Washington State University, Spokane, WA 99202, USA; solmaz.amiri@wsu.edu (S.A.); p.monsivais@wsu.edu (P.M.); 3 Department of Sociology, Washington State University, Pullman, WA 99164, USA; justin.denney@wsu.ed; 4 College of Public Health and Human Sciences, Oregon State University, Corvallis, OR 97331, USA; Perry.Hystad@oregonstate.edu; 5 Paul G. Allen School for Global Animal Health, Washington State University, Pullman, WA 99164, USA

**Keywords:** Parkinson’s disease, pesticide exposure, premature mortality, spatial analyses, Washington State

## Abstract

The aim of this study was to examine the relationship between estimated residential exposure to agricultural chemical application and premature mortality from Parkinson’s disease (PD) in Washington State. Washington State mortality records for 2011–2015 were geocoded using residential addresses, and classified as having exposure to agricultural land-use within 1000 meters. Generalized linear models were used to explore the association between land-use associated with agricultural chemical application and premature mortality from PD. Individuals exposed to land-use associated with glyphosate had 33% higher odds of premature mortality than those that were not exposed (Odds Ratio (OR) = 1.33, 95% Confidence Intervals (CI) = 1.06–1.67). Exposure to cropland associated with all pesticide application (OR = 1.19, 95% CI = 0.98–1.44) or Paraquat application (OR = 1.22, 95% CI = 0.99–1.51) was not significantly associated with premature mortality from PD, but the effect size was in the hypothesized direction. No significant associations were observed between exposure to Atrazine (OR = 1.21, 95% CI = 0.84–1.74) or Diazinon (OR = 1.07, 95% CI = 0.85–1.34), and premature mortality from PD. The relationship between pesticide exposure and premature mortality aligns with previous biological, toxicological, and epidemiological findings. Glyphosate, the world’s most heavily applied herbicide, and an active ingredient in Roundup^®^ and Paraquat, a toxic herbicide, has shown to be associated with the odds of premature mortality from PD.

## 1. Introduction

Parkinson’s disease (PD) is the second most prevalent neurological disorder in the United States, and is designated as a global public health challenge by the World Health Organization [1]. PD is a neurodegenerative disease that impacts most adults after the age of 50, increasing with age and reaching a heightened risk at roughly 80 years of age [2]. The chronic symptoms of PD are the foundation of the uncharacteristically high burden of disease in the United States, as the direct cost to care for PD patients was estimated at $14.4 billion in 2010. The indirect costs that are associated with PD are also high, as decreased productivity in younger individuals with PD has been estimated to equate to a $1.7 billion loss [3].

PD that is diagnosed before the age of 65 is considered to be younger-onset [4,5], and an earlier onset of PD was found to be associated with environmental exposures, such as the consumption of well-water [6]. Other environmental exposures associated with risk of PD include agricultural chemical exposure, residing in a rural location, and well-water contamination [5,7,8,9,10,11]. Washington State has a large agricultural economy, ranking first in the nation for production of apples, potatoes, wheat, cherries, hay, hops, and pears; all of which require a considerable chemical input for their production [12]. The state’s agriculture is highly concentrated in the Eastern side of the state, due to its climate and access to irrigation by the Grand Coulee Dam. Correspondingly, PD rates in Washington State are among the highest in the nation, and are 14 percent greater than the national average [13].

Previous epidemiological research on the state’s prevalence of PD in relation to agricultural chemical exposure was inconclusive. A population-based case control study in Western Washington yielded a non-significant, positive relationship between PD and occupational exposure to parathion, an agricultural pesticide that is no longer used in the United States [14]. A second study conducted in Washington State reported an increased risk of Parkinsonism in orchardists with the highest levels of exposure to agricultural pesticides. Failing to identify any culpable pesticides, that study was limited in that it relied on the participants’ recall to estimate pesticide exposures over the 40-year period (1972–2001) [10].

While previous studies concerning the relationship between environmental exposures and PD provide congruent results, many submissions fail to identify individual pesticides that have been shown to contribute to PD, and they often rely on the participants’ memory of pesticide application, leaving results subject to recall bias. This study aimed to examine the relationship between residential agriculturally-related exposures and PD-related mortality in Washington State. The study utilized advanced spatial analysis methods to combine routine geospatial data on agricultural land-use with accurate residential addresses to estimate and model individuals’ exposure to agricultural chemicals Atrazine, Diazinon, Glyphosate, and Paraquat, and premature mortality attributed to PD.

## 2. Materials and Methods

### 2.1. Data Sources

Registered deaths for the years 2011–2015 were obtained from the Washington State Department of Health, Center for Health Statistics [12]. The data file provided information regarding the individuals’ sex, race, education, marital status, occupation, residential latitude and longitude, underlying cause of death, and associated International Classification of Diseases [ICD-10] codes. Data were queried to include those whose deaths were classified as a direct or underlying cause of PD [ICD-10 G20]. Similar to previous studies, we used individual-level characteristics as covariates in the analysis [15]. Pesticide application land use classifications were obtained from the United States Geological Survey’s National Water-Quality Assessment Project (USGS NAWQA) for the same period [16]. Agricultural chemicals were chosen based on their prevalence in the State of Washington, their geographic range of application, and their presence in previous research in correlation to neurodegenerative symptoms such as PD [15,17,18,19]. Spatial cropland cover data were obtained from the United States Department of Agriculture’s (USDA) National Agricultural Statistics Service (NASS) Geospatial Information Branch (GIB) for the years of 2011–2015 [20]. Residential well-water locations were obtained using geospatial information from the Washington Department of Ecology’s Well Report [21].

This research did not use living human subjects, nor experimental animals. Because all subjects in this study were deceased, it did not require review or approval by a constituted committee for human subjects or animal research.

### 2.2. Measures

#### 2.2.1. Outcome Variable

The outcome of interest was premature mortality, defined as being less than 75 years of age at the time of death (yes, no). This measure was chosen for two reasons. First, we used the definition for older-age onset (>65 years), earlier-onset (≤65 years) [4], and the average life expectancy upon diagnosis of PD (9.1 years) [5]. Thus, a person who had been diagnosed after 65 years of age was expected to live to 75 years of age or greater, and vice versa. Second, the age of 75 was chosen because it acts as a good representation of the United States life expectancy, and it recognizes the prevalence of a chronic disease that occurs later in life. It also has been used in multiple studies as an indicator of disparities among populations [22,23].

#### 2.2.2. Exposure Variables

Exposure to agricultural pesticides was estimated using spatial analyses and a crop-exposure matrix that was applied to agricultural land around participants’ residential addresses (drift exposure), well locations (potentially hazardous well water consumption), and occupational exposure classifications from vital statistics (occupational exposure). All analyses were conducted with ESRI ArcGIS, v.10.6 (ESRI, Redlands, CA, USA) [24].

Cropland data for the State of Washington from 2011–2015 were reclassified to align with the NAWQA crop application groups into six categories of (a) alfalfa, (b) corn, (c) orchards and grapes, (d) pasture and hay, (e) soybeans, and (f) wheat. NAWQA uses these crop group classifications because they align with the USDA crop organizational structure [20]. Reclassified cropland data for the five years of interest were summed to create a single layer of agricultural land use within the given period for each type of cropland, yielding a total of six cropland layers.

To estimate the exposure to pesticide application, the following spatial procedure was conducted. Reclassified cropland data were converted from a raster to a vector format. Crop polygons of less than 18,000 m^2^ (4.4 acres) were removed because previous studies on geographic analysis of agricultural field size that have shown crop sizes smaller than this are likely be digitization errors [25]. Cropland use that aligned with the application of an individual pesticide according to the NAWQA classifications were combined to form a unique layer that represented the likely application of each agricultural chemical, yielding four individual crop-exposure layers. Residential addresses at the time of death were geocoded using ArcGIS, buffered by 1000 meters, and intersected with reclassified crop-exposure data to represent potential exposure to agricultural chemical application (ESRI, Redlands, CA, USA). We classified each address into a dichotomous exposure potential, rather than a continuous measure because of the effervescence of rotational crop pesticide application, and the likelihood of pesticide drift, measuring up to 1000 meters [19,26]. Measures were repeated for each individual with the three remaining pesticides. 

Potential well-water-based exposures were estimated by considering the wells’ proximity to cropland, and the plausible groundwater compromise to within 500 meters of cropland [15]. Wells were restricted to those classified as residential. The residential wells were further filtered to include the nearest wells to within 250 m of PD deaths, and assigned to each individual death. A dichotomous variable was developed by using the assigned wells that fell within 250 m of the deceased individual, and whether the drill site was within 500 m of agricultural cropland (yes, no).

The listed occupation that was associated with each of the deceased was used to estimate the likelihood of occupational exposure, using the industrial codes of the Bureau of Labor Statistics, classified as ‘Agriculture, Forestry, Fishing and Hunting’ (NAICS 11), with occupations categorized under ‘Crop Production’ (NAICS 111). A dichotomous variable was developed to show deaths that were associated with agricultural occupation, and thus, elevated agricultural chemical exposure (yes, no).

### 2.3. Demographic Variables

Demographic variables were defined as sex (male, female), race (white, non-white), marital status (two categories: married or living with domestic partner versus never married, divorced, separated, or widowed), and education (four categories: no high school diploma, high school graduate or equivalent, some college, and associate’s degree and or above).

### 2.4. Statistical Analysis

Univariate analyses included the reporting measure of central tendency and variability for continuous variables and frequency distributions, and percentages for categorical variables. Bivariate statistics included chi-square and the Mann–Whitney U to test for differences in demographic and exposure variables in the premature and non-premature groups. Multivariate analyses included generalized linear models (GLMs) with a binary logistic function to explore the association between exposure and premature mortality, controlling for covariates. Associations were presented as odds ratios (ORs) with 95% confidence intervals (CIs). Models were adjusted for sex, race, education, and marital status. SPSS v.25 (IBM, Armonk, NY, USA) was used to perform statistical modeling procedures, using a significance level of <0.05.

A sensitivity analysis was conducted by exploring the relationship between chemical exposure and mortality, with the underlying cause of stroke (ICD-10, I63) that occurred in Washington State during the same period. Stroke was used as a comparison, because the biological pathway of cerebral infarction is not induced by pesticide exposure [27].

### 2.5. Mapping

Maps were created by using the premature mortalities attributed to PD and spatial land-use data associated with glyphosate and Paraquat application. While the actual points were removed to conceal residential addresses, clusters with a high ratio of premature deaths that fell within 1000 m of glyphosate and/or Paraquat application to the total number of premature deaths were identified. 

## 3. Results

The characteristics of individuals who experienced premature mortality are shown in Table 1. A total of 4591 PD-related deaths between 2011 and 2015 were recorded (1.75 % of all deaths), of which 659 (14%) were premature. For all deaths attributed to PD, 94% were non-Hispanic white, and 62% were male. The median age at the time of death was 83 for all deaths with an underlying cause of PD. Approximately, 93% of those who died prematurely were non-Hispanic white, and 71% were male. Those who died prematurely had an average age of 71 years (versus 85 for non-premature death).

In the multivariable-adjusted models, the residential exposure to pesticide that was associated with all cropland was not significantly related to premature mortality by PD, but the OR was in the hypothesized direction (OR = 1.19, 95% CI = 0.98–1.44). The residential exposure to agricultural land use associated with glyphosate had 33% higher odds of premature mortality than those who were not exposed (OR = 1.33, 95% CI = 1.06–1.67). Exposure to cropland associated with Paraquat application was not significantly associated with premature mortality by PD, but the effect size was large and in the hypothesized direction (OR = 1.22, 95% CI = 0.99–1.51). No significant associations were observed between exposure to Atrazine (OR = 1.21, 95% CI = 0.84–1.74) or Diazinon (OR = 1.07, 95% CI = 0.85–1.34), and premature mortality by PD (Table 2). 

The sensitivity analysis showed insignificant associations between exposure to cropland, associated with all four agricultural chemicals, and premature mortality with the underlying cause of stroke (results are not shown).

**Figure 1:**
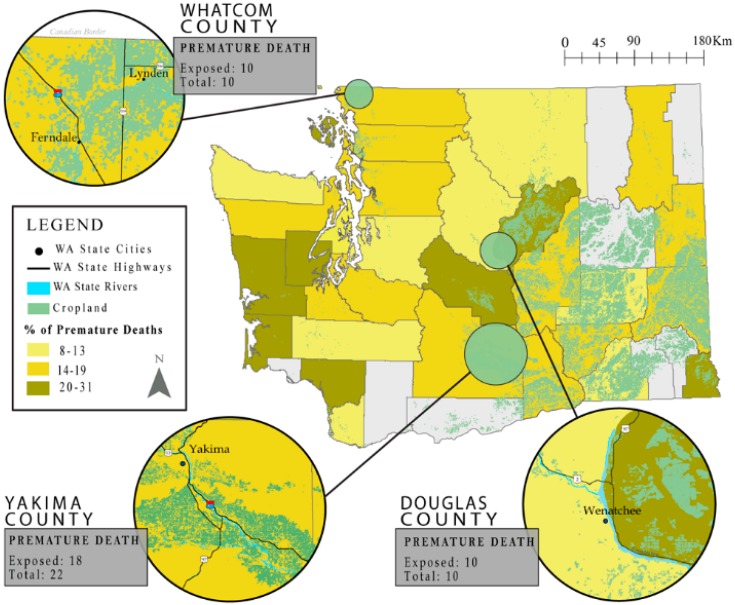
Percent premature death (659 of 4591 total deaths) at the county level, with inset maps of geographic areas that presented a high spatial correlation between the premature mortality and proximity to glyphosate and/or Paraquat application. Ratios can be described as the number of premature deaths within 1000 meters of application, divided by the total number of premature deaths. Grayed counties were excluded from analysis due to a small cell count.

When considering premature mortality on an individual level, several clusters of premature deaths were found in highly agricultural areas. A clear relationship was found in Douglas County, where the rates of premature deaths attributed to PD were the highest in the state (31%). A sizable clustering of premature deaths was found near the Northwestern border of the county. Within this cluster, all premature deaths that occurred in the area fell within 1000 m of glyphosate and/or Paraquat application. The proportion of deaths in Yakima valley was similarly indicative, with 18 of the 22 premature deaths falling within 1000 m of glyphosate and/or Paraquat application. The Yakima Valley shares similar agricultural productivity to Douglas County, making its inhabitants highly subject to both Paraquat and glyphosate exposures (Figure 1).

## 4. Discussion

This study utilized a large sample size to highlight the relationship between the residential exposure to agricultural pesticides, and the premature mortality associated with PD. Significant associations were found between exposure to glyphosate application and premature mortality associated with PD. Exposure to Paraquat was not significantly associated with premature mortality, but the effect was in the hypothesized direction. The relationship between Atrazine or Diazinon and premature mortality from PD were not statistically significant.

The strong relationship between Glyphosate exposure and premature mortality attributed to (PD) in our study aligns with the results found in multiple studies. Toxicological studies have found glyphosate and glyphosate-based herbicides to negatively affect neural cells, resulting in oxidative damage [18,28]. A study conducted on immature rats using Roundup^®^, in which glyphosate is the active ingredient, found that exposure to glyphosate resulted in signaling and enzymatic changes, that may lead to affected uptake of hippocampal cells. The cell damage upon exposure was suggestive of a relationship between glyphosate and neurotoxicity [18].

**Figure 2:**
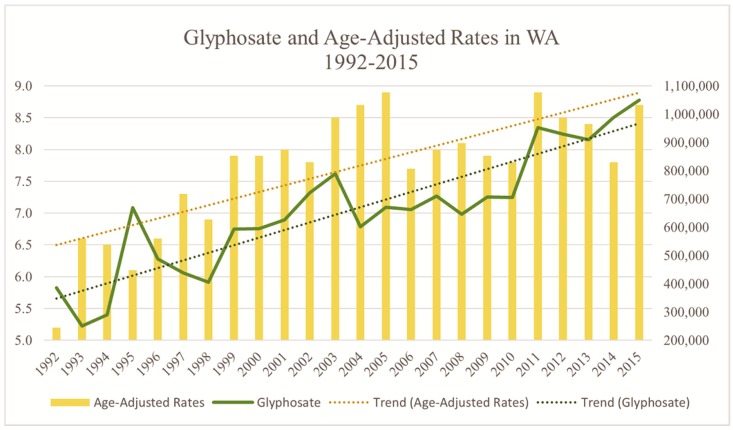
Relationship between glyphosate application and premature death by PD in the state of Washington from 1992–2015. The state’s age-adjusted rates of PD mortality rates have grown to be two-thirds greater in this time period, from 5.2 to 8.7. The age-adjusted rate at the release of Glyphosate in the late 1970s was 2.5 [Data Source: WSDH, USGS [14,29].

Roundup^®^ remains the most heavily applied herbicide in the world, with a similar trend that can be observed in Washington State. In the past 23 years, glyphosate application in the state of Washington has nearly tripled, while age-adjusted rates of PD have also risen (Figure 2).

Glyphosate exposures can trigger numerous biological effects that may progress before being made apparent in chronic degenerative diseases or other health problems [28]. Establishing the causal role of glyphosate in such chronic conditions is further complicated by the temporal relationship between pesticide exposure and adverse health outcomes. The effects following glyphosate-based herbicide exposures are not often immediately observed, but rather, they are caused by early-life exposure and they manifest in the later stages of adulthood, which is the perfect case to be made for those growing up and living in rural agricultural areas.

Although this study found exposure to Paraquat to be insignificantly associated with premature PD mortality, the associations were in the hypothesized direction, which is consistent with wider toxicological and epidemiologic literature. Previous studies most often cite Paraquat as a likely environmental neurotoxicant, because of its near identical molecular structure to MPTP (1-methyl-4-phenyl-1,2,3,6-tetrahydropyridine), whose neurotoxicity has been related to idiopathic PD [30]. A study conducted on rats found a dose-dependent relationship with Paraquat exposure and neuron loss, in which a slightly greater vulnerability was measured in older rats [31]. Epidemiologic studies have also found that exposure to Paraquat to increase risk of PD [19].

### Strengths and Limitations

This study is unique in multiple ways, with many innovative strengths and some implicit limitations. First, studies that relate agricultural chemical exposure and PD often implement a temporal model, in which researchers use the participants’ memory to recount the exposure of the study population. These studies are highly subject to recall bias. By analyzing data that is retrieved from PD-related deaths and by employing the advanced spatial methodologies described above, a much more detailed understanding of the individual exposure was developed, obviating the issues of participant recall bias. Further, agricultural chemical exposure assessments are greatly constricted by the historic pesticide records that are made available to researchers by federal institutions. A great deal of quantitative research on pesticide exposure and PD in the United States has been made possible by California’s independent Pesticide Usage Report (PUR), which offers extensive reports of agricultural chemical application in the state of California [8,15]. The USDA and USGS provide public records regarding pesticide application, but the highest resolution is provided on a county level, and only the USGS offers a dataset that is aggregated by specific chemical type from 1992 to 2015. While we acknowledge the limitation of using land-use classifications to estimate application, the USDA’s estimates for the crops that likely received specific pesticide application offered a much finer geographic estimation than the USDA’s county records were able to provide. To employ these records at a much greater resolution, highly comprehensive agricultural land-use data were needed, and unfortunately, the USDA has only made this spatial data available for the years of 2008 to 2017.

Although the agricultural chemical exposure analysis employed in this study did not allow for the temporal relationship of the exposure that predates symptoms of PD, historical geographic land-use analyses offered an important justification for why this technique was employed. A study that analyzed land cover change from 1950 to 2000 reported a boom of agricultural expansion in this region, which coincided with the development of irrigated agriculture in the 1950s [32]. Beside this period of expansion, agricultural land-use in the United States has declined due to a steady increase in agricultural mechanization and efficiency, albeit staying relatively steady in geographic areas such as the Western United States and the Midwest. Thus, there is good reason to believe that current farmland in Washington was highly likely to be farmland since the 1950s. Another consideration that authors considered when formulating these methods was the complexity of human mobility. Furthermore, we had only a single residential address that was associated with each death record, and had no way of knowing how long an individual that was affected by PD resided in the address where they last lived. We understand that this limitation is significant in considering the exposure estimation because an address cannot truly capture the complexities of pesticide exposure. Nonetheless, our choice in using residential records as an estimation for exposure has been shown to be meaningful to other exposure assessments [33,34,35].

Previous literature has associated early-onset PD with well-water consumption, which is suggestive of exposure from well-water sources. Glyphosate has been found to leach and contaminate groundwater, and its newly recognized half-life in soil and water varies greatly by geographic location. This chemical half-life can range from a few days to a year, and it depends greatly on soil composition, resulting in a highly variable and long-term buildup of glyphosate. The inherent specificity makes for costly testing and monitoring programs [36]. This site specificity may have been to blame for the insignificant results in our geographic analysis of well exposures. Lastly, the relationship between occupational exposure and premature mortality was statistically insignificant, which was likely due to the occupational classifications given on death certificates. The number of individuals who had died from PD and that were employed in the agricultural industry was only 112, although Washington State has been estimated to employ roughly 18,000 workers in the agricultural industry annually [37]. This small proportion of agricultural workers may be due to the misclassification of occupation. Over one half of farmers in Washington State are employed part-time in addition to other forms of employment; thus, death certificate employment records may have not fully encompassed this relationship, resulting in a smaller sample size and insignificant relationships [12].

While these findings have provided consistent results that align with previous toxicological, biological, and epidemiological findings, further research is required. In order to circumvent negative health outcomes, the following individual measures can be taken. First, the environmental risks of exposure to glyphosate should be made public knowledge, as should the location and timing of application in agricultural communities. As our research suggests, residents should also consider distancing themselves from agricultural cropland when making residential decisions. 

Finally, to perform additional research that questions the associations between agricultural chemical exposure and environmental health, institutional commitments to data collection will need to be reconsidered. Pesticide application collection and distribution should look to California’s Pesticide Use Reporting (PUR) collection, to begin collecting more comprehensive information such as date, time, location, field size, crop type, application type, pesticide type, and amount of pesticide applied. National health examination databases such as the United States Center for Disease Control and Prevention’s National Health and Nutrition Examination Survey (US CDC, NHANES) should work to improve the biomonitoring of pesticide accumulation in the tissues and bodily fluids of human populations, considering that pesticide residue can be found in food products, consumable well-water, and soil surfaces alike. Epidemiological studies would benefit greatly from a continuous database of chemical bioaccumulation, because the greatest limitations among studies are those relating to the temporality of pesticide-related illnesses. 

## 5. Conclusions

The positive relationship between pesticide exposure and premature mortality aligns with previous biological, toxicological, and epidemiological findings. Glyphosate, the world’s most heavily applied herbicide and active ingredient in Roundup^®^, and Paraquat, a toxic herbicide, were shown to be associated with the odds of premature mortality from PD. Especially relevant to the recent concern in glyphosate exposure, it is our hope that this work contributes to the wider discussion on the need to amend current pesticide regulations and public health policies. The United States Environmental Protection Agency’s (US EPA) regulations on glyphosate application are based on a risk assessment process that was conducted over 30 years ago [38]. Of concern, only 11 of the sources cited in this decision were peer-reviewed, which means that the regulation of the nation’s heaviest applied herbicide is still reliant on research conducted decades ago [39]. The United States Federal Insecticide, Fungicide, and Rodenticide Act (FIFRA) insists that a pesticide may not be applied unless it can be shown to not have “any unreasonable risk to man or the environment, taking into account the economic, social, and environmental costs and benefits of any pesticide” [40]. It is a shared concern that advocates for glyphosate application rely on outdated risk assessments, and the lack of concrete data collection among those exposed, to validate their application of the pesticide [39]. While we understand the significance of glyphosate in US agriculture, our research suggests that it will become increasingly important to consider the human and environmental costs of glyphosate application.

## Figures and Tables

**Table 1:** Demographic characteristics of individuals who died from Parkinson’s disease from 2011–2015.

Characteristics	Total	Premature Mortality (Age ≤ 74 Years)	Non-Premature Mortality (Age > 75 Years)	*p*-Value
	(n = 4591)	(n = 659)	(n = 3932)	
Age (median, IQR)	83 (78–88)	71 (67–72)	85 (81–89)	<0.001
Sex (no. (%))				
Female	1744	189 (28.7)	1555 (39.5)	<0.001
Male	2847	470 (71.3)	2377 (60.5)	
Race/ethnicity (no. (%))				
Other	274	45 (6.8)	229 (5.8)	0.31
Non-Hispanic white	4311	612 (92.9)	3699 (94.1)	
Unknown	6	2 (0.3)	4 (0.1)	
Education (no. (%))				
Less than High School Diploma	587	66 (10.0)	521 (13.3)	<0.001
High School Diploma or equivalent	1573	191 (29.0)	1382 (35.1)	
Some college	1025	185 (28.1)	840 (21.4)	
Associate’s Degree or higher	1399	216 (32.8)	1183 (30.1)	
Unknown	7	1 (0.1)	6 (0.1)	
Marital status (no. (%))				
Married or living with domestic partner	2243	399 (60.5)	1844 (46.9)	<0.001
Never married, divorced, separated, or widowed	2348	260 (39.5)	2088 (53.1)	
Likelihood of occupational exposure (no. (%))				
Likely	112	11 (1.7)	101 (2.6)	0.17
Unlikely	4479	648 (98.3)	3831 (97.4)	
Likelihood of well-water intake (no. (%))				
Likely	295	52 (18.1)	243 (81.9)	0.10
Unlikely	4296	607 (14.2)	3689 (85.8)	

Notes: IQR = Interquartile range; no = number.

**Table 2:** Unadjusted and adjusted odds ratios of premature mortality by PD from exposure to individual agricultural chemicals.

Agricultural Chemical	N	Unadjusted OR (95%CI)	*p*-Value	N	Adjusted OR (95%CI)	*p*-Value
All Pesticide						
No Exposure	3526	Reference		3516	Reference	
Exposed	1065	1.16 (0.96–1.40)	0.13	1062	1.19 (0.98–1.44)	0.09
Atrazine						
No Exposure	4357	Reference		4344	Reference	
Exposed	234	1.17 (0.82–1.67)	0.40	231	1.21 (0.84–1.74)	0.31
Diazinon						
No Exposure	3833	Reference		3821	Reference	
Exposed	758	1.06 (0.85–1.31)	0.63	757	1.07 (0.85–1.34)	0.63
Glyphosate						
No Exposure	3932	Reference		3914	Reference	
Exposed	659	1.30 (1.04–1.62)	0.02	664	1.33 (1.06–1.67)	0.01
Paraquat						
No Exposure	3780	Reference		3769	Reference	
Exposed	811	1.20 (0.98–1.48)	0.09	809	1.22 (0.99–1.51)	0.07

Notes: OR = Odds Ratio; CI = Confidence Interval.

## References

[1] Baker M., Gershanik O., Aarli J.A., Dua T., Janca A., Muscetta A. (2006). Parkinson’s disease. Neurological Disorders: Public Health Challenges.

[2] Bower J.H., Maraganore D.M., McDonnell S.K., Rocca W.A. (2000). Influence of strict, intermediate, and broad diagnostic criteria on the age- and sex-specific incidence of Parkinson’s disease. Mov. Disord..

[3] Kowal S.L., Dall T.M., Chakrabarti R., Storm M.V., Jain A. (2013). The current and projected economic burden of Parkinson’s disease in the United States. Mov. Disord..

[4] Ishihara L.S., Cheesbrough A., Brayne C., Schrag A. (2007). Estimated life expectancy of Parkinson’s patients compared with the UK population. J. Neurol. Neurosurg. Psychiatry.

[5] Delamarre A., Meissner W.G. (2017). Epidemiology, environmental risk factors and genetics of Parkinson’s disease. La Presse Médicale.

[6] Tsai C.H., Lo S.K., See L.C., Chen H.Z., Chen R.S., Weng Y.H., Chang F.C., Lu C.S. (2002). Environmental risk factors of young onset Parkinson’s disease: A case-control study. Clin. Neurol. Neurosurg..

[7] Ritz B., Costello S. (2006). Geographic model and biomarker-derived measures of pesticide exposure and Parkinson’s disease. Ann. N. Y. Acad. Sci..

[8] Wang A., Costello S., Cockburn M., Zhang X., Bronstein J., Ritz B. (2011). Parkinson’s disease risk from ambient exposure to pesticides. Eur. J. Epidemiol..

[9] van der Mark M., Brouwer M., Kromhout H., Nijssen P., Huss A., Vermeulen R. (2012). Is pesticide use related to Parkinson disease? Some clues to heterogeneity in study results. Environ. Health Perspect..

[10] Engel L.S., Checkoway H., Keifer M.C., Seixas N.S., Longstreth W.T., Scott K.C., Hudnell K., Anger W.K., Camicioli R. (2001). Parkinsonism and occupational exposure to pesticides. Occup. Environ. Med..

[11] Firestone J.A., Smith-Weller T., Franklin G., Swanson P., Longstreth W.T., Checkoway H. (2005). Pesticides and risk of Parkinson disease: A population-based case-control study. Arch. Neurol..

[12] Washington State Department of Agriculture Washington Agriculture: A Global Impact. https://agr.wa.gov/aginwa/default.aspx.

[13] Centers for Disease Control and Prevention Parkinson’s Disease Mortality by State. https://www.cdc.gov/nchs/pressroom/sosmap/parkinsons_disease_mortality/parkinsons_disease.htm.

[14] Firestone J.A., Lundin J.I., Powers K.M., Smith-Weller T., Franklin G.M., Swanson P.D., Longstreth W.T., Checkoway H. (2010). Occupational factors and risk of Parkinson’s disease: A population-based case-control study. Am. J. Ind. Med..

[15] Gatto N.M., Cockburn M., Bronstein J., Manthripragada A.D., Ritz B. (2009). Well-water consumption and Parkinson’s disease in rural California. Environ. Health Perspect..

[16] Baker N.T., Stone W.W. (2015). Estimated Annual Agricultural Pesticide Use for Counties of the Conterminous United States, 2008–2012.

[17] Filipov N.M., Stewart M.A., Carr R.L., Sistrunk S.C. (2007). Dopaminergic toxicity of the herbicide atrazine in rat striatal slices. Toxicology.

[18] Cattani D., de Liz Oliveira Cavalli V.L., Heinz Rieg C.E., Domingues J.T., Dal-Cim T., Tasca C.I., Mena Barreto Silva F.R., Zamoner A. (2014). Mechanisms underlying the neurotoxicity induced by glyphosate-based herbicide in immature rat hippocampus: Involvement of glutamate excitotoxicity. Toxicology.

[19] Costello S., Cockburn M., Bronstein J., Zhang X., Ritz B. (2009). Parkinson’s disease and residential exposure to maneb and paraquat from agricultural applications in the central valley of California. Am. J. Epidemiol..

[20] United States Department of Agriculture Washington Cropland Data Layer. https://www.nass.usda.gov/Research_and_Science/Cropland/SARS1a.php.

[21] Washington State Department of Ecology Well Reports. https://fortress.wa.gov/ecy/wellconstruction/map/wclswebMap/default.aspx.

[22] Chen J.T., Rehkopf D.H., Waterman P.D., Subramanian S.V., Coull B.A., Cohen B., Ostrem M., Krieger N. (2006). Mapping and measuring social disparities in premature mortality: The impact of census tract poverty within and across Boston neighborhoods, 1999–2001. J. Urban Health.

[23] Mansfield C.J., Wilson J.L., Kobrinski E.J., Mitchell J. (1999). Premature mortality in the United States: The roles of geographic area, socioeconomic status, household type, and availability of medical care. Am. J. Public Health.

[24] ESRI (2018). ArcGIS Desktop.

[25] Yan L., Roy D.P. (2016). Conterminous United States crop field size quantification from multi-temporal Landsat data. Remote Sens. Environ..

[26] Ward M.H., Lubin J., Giglierano J., Colt J.S., Wolter C., Bekiroglu N., Camann D., Hartge P., Nuckols J.R. (2006). Proximity to crops and residential exposure to agricultural herbicides in Iowa. Environ. Health Perspect..

[27] Warlow C.P. (1998). Epidemiology of stroke. Lancet.

[28] Myers J.P., Antoniou M.N., Blumberg B., Carroll L., Colborn T., Everett L.G., Hansen M., Landrigan P.J., Lanphear B.P., Mesnage R. (2016). Concerns over use of glyphosate-based herbicides and risks associated with exposures: A consensus statement. Environ. Health.

[29] Washington State Department of Health Services Center for Health Statistic Death Data. https://www.doh.wa.gov/DataandStatisticalReports/HealthStatistics/Death.

[30] Snyder H.S., D’amato J.R. (1986). MPTP: A neurotoxin relevant to the pathophysiology of Parkinson’s disease: The 1985 George C. Cotzias Lecture. Neurology.

[31] McCormack A.L., Thiruchelvam M., Manning-Bog A.B., Thiffault C., Langston J.W., Cory-Slechta D.A., Di Monte D.A. (2002). Environmental risk factors and Parkinson’s disease: Selective degeneration of nigral dopaminergic neurons caused by the herbicide paraquat. Neurobiol. Dis..

[32] Brown D.G., Johnson K.M., Loveland T.R., Theobald D.M. (2005). Rural Land-Use Trends in the Conterminous United States, 1950–2000. Ecol. Appl..

[33] Gunier R.B., Bradman A., Harley K.G., Kogut K., Eskenazi B. (2017). Prenatal Residential Proximity to Agricultural Pesticide Use and IQ in 7-Year-Old Children. Environ. Health Perspect..

[34] Harnly M., McLaughlin R., Bradman A., Anderson M., Gunier R. (2005). Correlating agricultural use of organophosphates with outdoor air concentrations: A particular concern for children. Environ. Health Perspect..

[35] Harnly M.E., Bradman A., Nishioka M., McKone T.E., Smith D., McLaughlin R., Kavanagh-Baird G., Castorina R., Eskenazi B. (2009). Pesticides in dust from homes in an agricultural area. Environ. Sci. Technol..

[36] Szekacs A., Darvas B. (2012). Forty years with Glyphosate. Herbicide Properties, Synthesis and Control of Weeds.

[37] Bureau of Labor Statistics Washington State Occupational Employment Statistics. https://esd.wa.gov/labormarketinfo/occupations.

[38] EPA (1993). Reregistration Eligibility Decision (RED) for Glyphosate.

[39] Vandenberg L.N., Blumberg B., Antoniou M.N., Benbrook C.M., Carroll L., Colborn T., Everett L.G., Hansen M., Landrigan P.J., Lanphear B.P. (2017). Is it time to reassess current safety standards for glyphosate-based herbicides?. J. Epidemiol. Community Health.

[40] EPA (1996). Federal Insecticide, Fungicide, and Rodenticide Act.

